# A robust twelve-gene signature for prognosis prediction of hepatocellular carcinoma

**DOI:** 10.1186/s12935-020-01294-9

**Published:** 2020-06-03

**Authors:** Guoqing Ouyang, Bin Yi, Guangdong Pan, Xiang Chen

**Affiliations:** 1 Department of Hepatobiliary Surgery, Liuzhou People’s Hospital, Liuzhou, China; 2 Department of Cardio-Vascular Surgery, Sun Yat-sen Memorial Hospital, Sun Yat-sen University, Guangzhou, China; 3 Department of General Surgery, The Second Xiangya Hospital, Central South University, Changsha, China

**Keywords:** Hepatocellular carcinoma, Overall survival, The Cancer Genome Atlas, Gene signature, Prognosis, DNA methylation

## Abstract

**Background:**

The prognosis of hepatocellular carcinoma (HCC) patients remains poor. Identifying prognostic markers to stratify HCC patients might help to improve their outcomes.

**Methods:**

Six gene expression profiles (GSE121248, GSE84402, GSE65372, GSE51401, GSE45267 and GSE14520) were obtained for differentially expressed genes (DEGs) analysis between HCC tissues and non-tumor tissues. To identify the prognostic genes and establish risk score model, univariable Cox regression survival analysis and Lasso-penalized Cox regression analysis were performed based on the integrated DEGs by robust rank aggregation method. Then Kaplan–Meier and time-dependent receiver operating characteristic (ROC) curves were generated to validate the prognostic performance of risk score in training datasets and validation datasets. Multivariable Cox regression analysis was used to identify independent prognostic factors in liver cancer. A prognostic nomogram was constructed based on The Cancer Genome Atlas (TCGA) dataset. Finally, the correlation between DNA methylation and prognosis-related genes was analyzed.

**Results:**

A twelve-gene signature including SPP1, KIF20A, HMMR, TPX2, LAPTM4B, TTK, MAGEA6, ANX10, LECT2, CYP2C9, RDH16 and LCAT was identified, and risk score was calculated by corresponding coefficients. The risk score model showed a strong diagnosis performance to distinguish HCC from normal samples. The HCC patients were stratified into high-risk and low-risk group based on the cutoff value of risk score. The Kaplan–Meier survival curves revealed significantly favorable overall survival in groups with lower risk score (P < 0.0001). Time-dependent ROC analysis showed well prognostic performance of the twelve-gene signature, which was comparable or superior to AJCC stage at predicting 1-, 3-, and 5-year overall survival. In addition, the twelve-gene signature was independent with other clinical factors and performed better in predicting overall survival after combining with age and AJCC stage by nomogram. Moreover, most of the prognostic twelve genes were negatively correlated with DNA methylation in HCC tissues, which SPP1 and LCAT were identified as the DNA methylation-driven genes.

**Conclusions:**

We identified a twelve-gene signature as a robust marker with great potential for clinical application in risk stratification and overall survival prediction in HCC patients.

## Background

Hepatocellular carcinoma (HCC) is one of the most common malignant solid tumors and the fourth leading cause of cancer-related deaths worldwide [1]. There were approximately 841,000 new cases of HCC and 782,000 deaths in 2018. The incidence and mortality continue to increase for both sexes. Surgical resection is the most effective treatment to cure early stage HCC, but most patients are first diagnosed at an advanced stage which missed the best time for surgical treatment. Although chemotherapy, radiotherapy, liver transplantation and other potentially curative treatment have achieved a variety of therapeutic effects and prolonged survival period, the prognosis of HCC remains poor due to the high rate of recurrence and intrahepatic spread [2]. Those high-risk HCC patients with potentially poor outcomes must be monitored and adopt timely and effective treatments to prolong survival and improve quality of life [3]. Therefore, it is an urgent need for effective prediction markers to accurately assess the prognosis of HCC patients.

Prognostic models based on parameters including clinical baseline characteristics to molecular biomarkers for HCC have been constructed in several previous studies [4]. A prognostic score by using positive tumor markers (alpha-fetoprotein (AFP), fucosylated AFP and des-gamma-carboxy prothrombin) showed a useful predictive prognostic value in HCC patients treated with transcatheter arterial chemoembolization [5]. But the characteristic of tumor markers, depended on tumor burden, limits their value in diagnosing early stage tumors. With the developments in gene chips and high-throughput sequencing, gene signature based on mRNA expression levels have shown great potential in predicting HCC prognosis. The abnormal expression levels of single gene such as SEC62 [6], SHP-1 [7], RING1 [8], AGBL2 [9] have been reported to be independent prognostic factors for HCC patients. Moreover, a risk‐coefficient model based on a multigene mRNA expression signature has been identified to be an independent prognostic factor for overall survival (OS) and could stratify patients into high- and low-risk group with significantly different OS [10–12]. These gene signatures could be used for the preclinical and clinical treatment for HCC patients. However, additional gene signatures are needed for accurate prognosis of HCC because of complexity and heterogeneity of this disease.

In the current study, six sets of differentially expressed genes (DEGs) from different Gene Expression Omnibus (GEO) datasets were integrated to identify overlapping DEGs. Functional annotation assessment with Gene Ontology (GO) annotation and Kyoto Encyclopedia of Genes and Genomes (KEGG) pathway enrichment analyses were conducted with the overlapping DEGs. Univariable and Lasso-Cox regression analysis were applied to identify overall survival-related DEGs and propose a prognostic risk score model to stratify HCC patients. Besides, independent prognostic factors of OS were identified by multivariable Cox survival analysis. We finally identified twelve-gene signature as a robust marker with great potential in risk stratification and OS prediction in HCC patients.

## Methods

### Study population

In present study, we searched and downloaded mRNA expression chip data of HCC tissues from the GEO database by using the keywords of “hepatocellular carcinoma” and “Homo sapiens”. Six microarray datasets (GSE121248, GSE84402, GSE65372, GSE51401, GSE45267 and GSE14520 (based on the GPL571 platform) were obtained for DEGs analysis. Details of the GEO datasets used in this study are shown in Table 1. RNA-sequencing data of 371 HCC tissues and 50 normal tissues normalized by log2 transformation were acquired from The Cancer Genome Atlas (TCGA) for analyzing the integrated DEGs from the six GEO datasets and building gene prognostic models. GSE14520 datasets (based on the GPL3921 platform) included 216 HCC tissues with complete clinical information and mRNA expression data for external validation of the prognostic gene signature. After excluding TCGA cases with incomplete clinical information, 233 HCC patients along with their complete age, gender, sex, tumor grade, American Joint Committee on Cancer (AJCC) pathologic tumor stage, vascular invasion, OS status and time information were included for univariable and multivariable Cox regression analysis. Mutation data were obtained from the cBioPortal for Cancer Genomics [13].

**Table 1:** Details of the GEO datasets included in this study

Datasets	References	Platform	Sample size (tumor/control)	Application
GSE121248	Hui et al.	GPL570 [HG-U133_Plus_2] Affymetrix Human Genome U133 Plus 2.0 Array	107 (70/37)	Identification of DEGs
GSE84402	Cheng et al.	GPL570 [HG-U133_Plus_2] Affymetrix Human Genome U133 Plus 2.0 Array	28 (14/14)	Identification of DEGs
GSE65372	Hoshida et al.	GPL14951 Illumina HumanHT-12 WG-DASL V4.0 R2 expression beadchip	54 (39/15)	Identification of DEGs
GSE51401	Kong et al.	GPL570 [HG-U133_Plus_2] Affymetrix Human Genome U133 Plus 2.0 Array	64 (30/34)	Identification of DEGs
GSE45267	Hsieh et al.	GPL570 [HG-U133_Plus_2] Affymetrix Human Genome U133 Plus 2.0 Array	87 (46/41)	Identification of DEGs
GSE14520	Wang et al.	GPL571 [HG-U133A_2] Affymetrix Human Genome U133A 2.0 Array	41 (22/19)	Identification of DEGs
GSE14520	Wang et al.	GPL3921 [HT_HG-U133A] Affymetrix HT Human Genome U133A Array	445 (225/220)	Validation of DEGs

### Processing of gene expression data

To integrated gene expression chip data downloaded from the GEO datasets, we firstly conducted background correction, quartile normalization for the raw data followed by log2 transformation to obtain normally distributed expression values. The DEGs between HCC tissues and non-tumor tissues were identified using the “Limma” package in R [14]. The thresholds of absolute value of the log2 fold change (logFC) > 1 and adjusted *P* value < 0.05 were adopted. Mean expression values were applied for genes with multiprobes. Then, we used the robust rank aggregation (RRA) method to finally identify overlapping DEGs (*P* < 0.05) from the six GEO datasets.

### Construction of a potential prognostic signature

To identify the prognostic genes, we firstly sifted 341 patients from the TCGA Liver Hepatocellular Carcinoma (TCGA-LIHC) cohort with follow-up times of more than 30 days. Then, univariable Cox regression survival analysis was performed based on the overlapping DEGs. A value of *P* < 0.01 in the univariable Cox regression analysis was considered statistically significant. Subsequently, the prognostic gene signature was constructed by Lasso‐penalized Cox regression analysis [15], and the optimal values of the penalty parameter alpha were determined through 10-times cross-validations by using R package “glmnet” [16]. Based on the optimal alpha value, a twelve-gene prognostic signature with corresponding coefficients was selected, and a risk score was calculated for each TCGA-LIHC patient. Next, the HCC patients were divided into two or three groups based on the optimal cutoff of the risk score determined by “survminer” package in R and X-Tile software. To assess the performance of the twelve-gene prognostic signature, the Kaplan–Meier estimator curves and the C-index comparing the predicted and observed OS were calculated using package “survival” in R. Time-dependent receiver operating characteristic (ROC) curve analysis was also conducted by using the R packages “pROC” [17] and “survivalROC” [18]. Then, the GSE14520 datasets with complete clinical information was used to validate the prognostic performance of twelve-gene signature. The GSE14520 external validation datasets was based on the GPL3921 platform of the Affymetrix HT Human Genome U133A Array Plate Set (HT_HG-U133A, Affymetrix, Santa Clara, CA, United States).

### Independence of the prognostic gene signature from other clinical parameters in TCGA

The risk score and other clinical variants, including age, body mass index (BMI), sex, tumor grade, the AJCC pathologic tumor stage, vascular invasion, residual tumor status and AFP value, were analyzed by univariable Cox regression analysis. Next, we conducted a multivariable Cox regression model that combined the risk score and the above clinical indicators (*P* value < 0.2) to assess the predictive performance. The univariable and multivariable Cox regression analysis were performed with TCGA-LIHC patients (n = 234) that had complete clinical information.

### Building and validating a predictive nomogram

A composite nomogram [19] was constructed based on all independent prognostic parameters previous screened to predict the probability of 1-year, 3-year and 5-year OS using the “rms” package in R software. The time-dependent area under the ROC curve (AUC) was calculated to determine the discriminatory ability of the above prognostic parameters. Then we used a calibration curve to visualize the performance of the nomogram with the observed rates at corresponding time points. Based on the total number of points of the nomogram, the patients were also stratified into two or three groups according to the optimal cutoffs. The Kaplan–Meier survival curves for the different groups were then plotted.

### Functional enrichment analysis of DEGs and prognosis-related genes

To detect potential biological functions and involved signaling pathways of DEGs, GO and KEGG enrichment analyses were performed by DAVID. Only *P* value < 0.05 was considered statistically significant. In addition, the protein‐protein interaction (PPI) network was applied to explore potential interactions mRNAs via the STRING database and was visualized via Cytoscape v.3.7.1.

### The correlation between DNA methylation and prognosis-related genes

The DNA methylation data of 371 HCC tissues and 50 normal tissues were acquired from TCGA database. The average DNA methylation beta-value for all CpG sites of a gene was calculated as the DNA methylation value for that gene. To examine the relationship between DNA methylation level and the corresponding mRNA expression value, the “MethylMix” package in R was utilized [20]. MethylMix identifies differential and functional DNA methylation by using a beta mixture model to identify tumor samples with different DNA methylation compared to normal tissue. Functional DNA methylation refers to a significant negative correlation between methylation and gene expression of a particular gene.

### Statistical analysis

R software 3.5.0 was used for all statistical analyses. Categorical variables were analyzed by the χ^2^ or Fisher’s exact test. Continuous variables were analyzed using Student’s t-test for paired samples. Two groups of boxplots were analyzed using the Wilcoxon-test. The heatmap of the DEGs was visualized using the “pheatmap” [21] package in R with zero-mean normalization. Kaplan‐Meier survival curves were built and hazard ratio (HR) with 95% confidence interval (CI) were calculated by log-rank tests. Correlations among the individual genes in the signature were assessed by Pearson correlation coefficients. All statistical tests were two-sided, *P* < 0.05 was considered statistically significant.

## Results

### Identification of DEGs

**Fig. 1:**
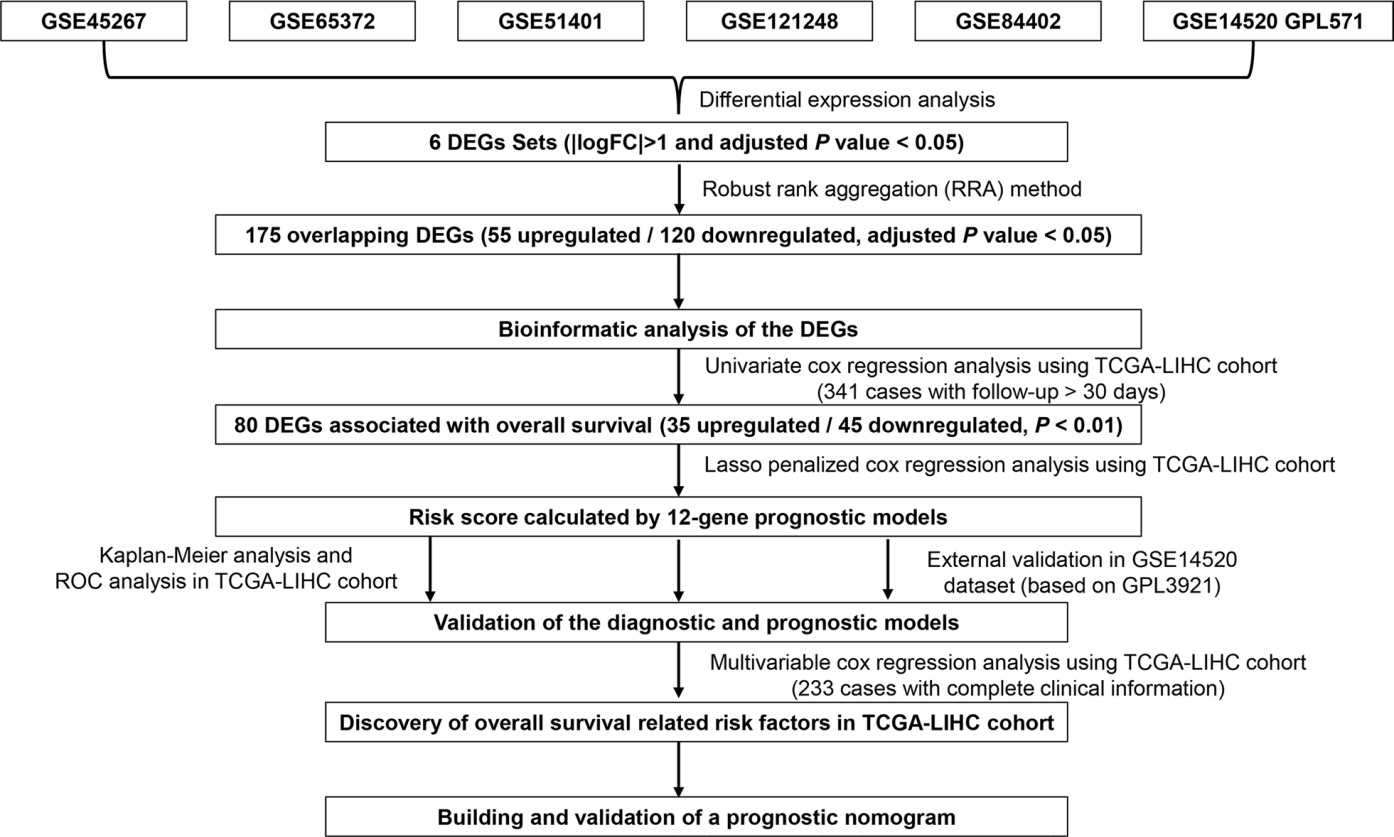
Flowchart describing the process used to identify and validate the prognostic gene signature of hepatocellular carcinoma

**Fig. 2:**
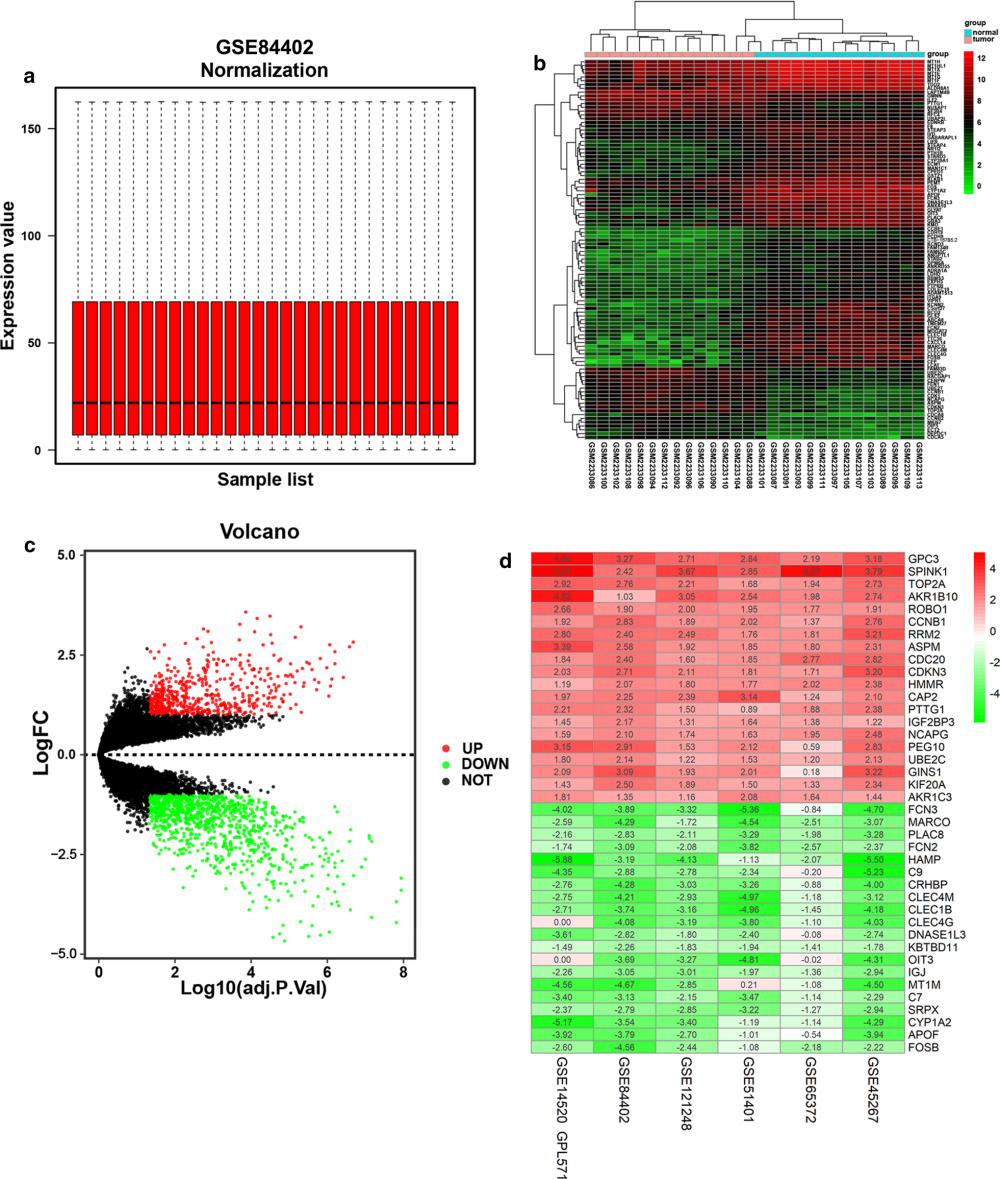
Identification of DEGs in liver cancer between tumor and normal tissues. **a** Normalization of GSE84402 dataset. **b** Representative heatmap of GSE84402 dataset shows that the DEGs can effectively differentiate tumors from normal tissues. **c** Volcano plot of GSE84402 dataset. **d** Heatmap of each expression microarray. The heat map of top 20 upregulated (red) and downregulated (green) DEGs identified by the robust rank aggregation method with the 6 GEO datasets. The value in each column represents the value of LogFC

The overall data processing workflow is shown in Fig. 1. Before identifying the DEGs, the six microarray datasets were normalized (Fig. 2a; Additional file 1: Figure S1A–F). As previously mentioned, details of the GEO datasets used in this study were presented in Table 1. Hierarchical clustering analysis demonstrated differences in DEGs expression patterns between tumor and non-tumor tissues (Fig. 2b; Additional file 1: Figure S1A–F). Volcano plots were generated to show the distribution of the DEGs (Fig. 2c; Additional file 1: Figure S1A–F). Next, we integrated the six sets of DEGs between tumor and non-tumor tissues by RRA method. Finally, a total of 175 overlapping DEGs, including 55 upregulated and 120 downregulated genes, were identified (Additional file 2: Table S1). The top 20 overlapping upregulated and downregulated DEGs in the six datasets are showed in Fig. 2d.

### Functional enrichment and PPI network analysis of the DEGs

**Fig. 3:**
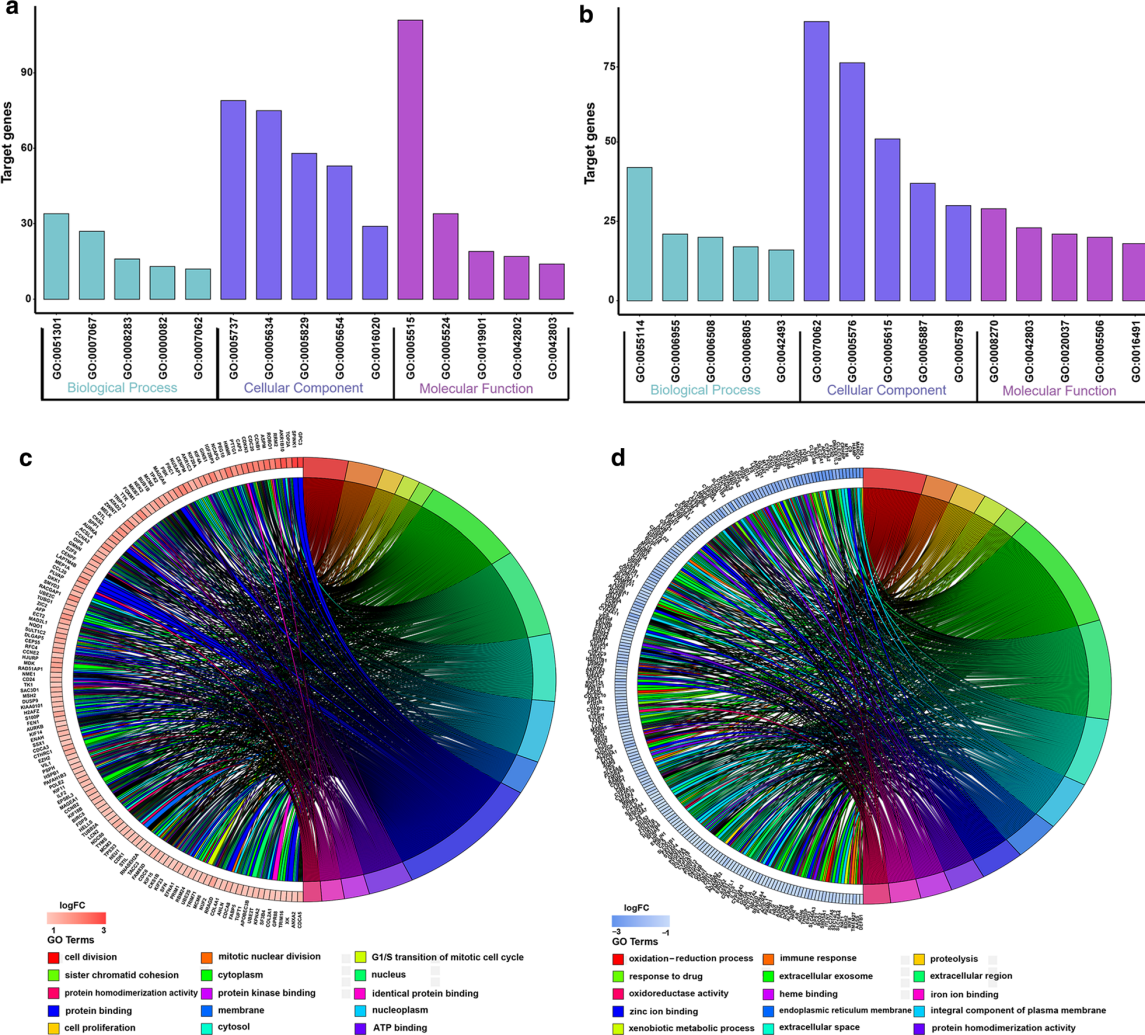
GO analysis of integrated DEGs in hepatocellular carcinoma. **a** Upregulated DEGs with the top 15 enriched GO terms. **b** Downregulated DEGs with the top 15 enriched GO terms. **c** Distribution of upregulated DEGs for different GO-enriched functions. **d** Distribution of downregulated DEGs for different GO-enriched functions

To further analyze biological information from the upregulated and downregulated overlapping DEGs (logFC > 1, *P* value < 0.05), GO enrichment analysis was performed respectively through the online DAVID tool. Concerning biological processes, the downregulated DEGs were significantly enriched in oxidation–reduction process, immune response and proteolysis. The upregulated DEGs were significantly enriched in cell division, mitotic nuclear division and G1/S transition of mitotic cell cycle. Enrichment analysis of cellular compartment and molecular functions and the corresponding distributions are shown in Fig. 3. KEGG pathway analysis showed that the DEGs were mainly enriched in metabolic pathways, chemical carcinogenesis, biosynthesis of antibiotics, retinol metabolism and cell cycle (Additional file 3: Figure S2A, Additional file 4: Table S2).

The PPI network of the DEGs was constructed by using the STRING database. A total of 400 nodes and 4238 edges were obtained and analyzed in the PPI network. Then, the top 25 candidate hub genes were identified with the cytoHubba plugin by using MCC method from Cytoscape (Figure S2B). Module analysis identified significant clustering modules in the PPI network. As illustrated in Figure S3A-C, the three highest-scoring functional clusters of modules were selected by the MCODE app (module 1, MCODE score = 70.421; module 2, MCODE score = 11.12; module 3, MCODE score = 6.121). KEGG pathways of each module were determined and are showed in Additional file 5: Figure S3.

### Identification of survival-related DEGs and establishment of the twelve-gene prognostic signature

A total of 341 TCGA-LIHC samples with a follow-up period > 30 days were included from 365 TCGA-LIHC patients for subsequent survival analyses. The baseline characteristics of these patients were listed in Table 2. Based on the univariable Cox regression model, 80 of total 175 DEGs were considered to be significantly correlated with OS, including 35 upregulated DEGs and 45 downregulated DEGs (*P* < 0.01). Then, Lasso penalized Cox regression analysis was performed, and a prognostic twelve-gene signature was identified, consisting of secreted phosphoprotein 1 (SPP1), annexin10 (ANXA10), leukocyte cell-derived chemotaxin 2 (LECT2), kinesin family member 20A (KIF20A), hyaluronan mediated motility receptor (HMMR), TTK, melanoma antigen A6 (MAGEA6), lysosomal protein transmembrane 4 beta (LAPTM4B), cytochrome P450 family 2 subfamily C member 9 (CYP2C9), retinol dehydrogenase 16 (RDH16), lecithin-cholesterol acyltransferase (LCAT) and targeting protein for xenopus kinesin-like protein 2 (TPX2) (Additional file 6: Figure S4, Additional file 7: Table S3).

**Table 2:** Baseline characteristics of TCGA-LIHC patients

Clinical features	Number (%)	Clinical features	Number (%)
Follow-up time (months)	26.7 ± 23.9	AJCC stage	
Survival status		Stage I	170 (46.6%)
Alive	235 (64.4%)	Stage II	84 (23.0%)
Dead	130 (35.6%)	Stage III	83 (22.7%)
Age	59.7 ± 13.4	Stage IV	4 (1.1%)
Sex		Not applicable	24 (6.6%)
Male	246 (67.4%)	T classification	
Female	119 (32.6%)	T1	180 (49.3%)
History of other malignancy		T2	91 (24.9%)
No	331 (90.7%)	T3	78 (21.4%)
Yes	34 (9.3%)	T4	13 (3.6%)
History of neoadjuvant treatment		TX	1 (0.3%)
No	363 (99.5%)	Not applicable	2 (0.5%)
Yes	2 (0.5%)	N classification	
History of radiation treatment		N0	248 (67.9%)
No	238 (65.2%)	N1	4 (1.1%)
Yes	4 (1.1%)	NX	112 (30.7%)
Not applicable	123 (33.7%)	Not applicable	1 (0.3%)
History of chemotherapy		M classification	
No	222 (60.8%)	M0	263 (72.1%)
Yes	14 (3.8%)	M1	3 (0.8%)
Not applicable	129 (35.3%)	MX	99 (27.1%)
History of ablation embolization		Grade	
No	230 (63.0%)	G1	55 (15.1%)
Yes	13 (3.6%)	G2	175 (47.9%)
Not applicable	122 (33.4%)	G3	118 (32.3%)
History of hepatic carcinoma risk factory		G4	12 (3.3%)
No history of primary risk factors	91 (24.9%)	Not applicable	5 (1.4%)
Alcohol consumption	115 (31.5%)	Vascular invasion	
Hepatitis B	102 (27.9%)	No	205 (56.2%)
Hepatitis C	55 (15.1%)	Micro	90 (24.7%)
Non-alcoholic fatty liver disease	19 (5.2%)	Macro	16 (4.4%)
Hemochromatosis	6 (1.6%)	Not applicable	54 (14.8%)
Other	30 (8.2%)	Relapse	
Not available	18 (4.9%)	No	172 (47.1%)
Residual tumor		Yes	94 (25.8%)
RO	320 (87.7%)	Not applicable	99 (27.1%)
R1	17 (4.7%)	Histological diagnosis	
R2	1 (0.3%)	Hepatocellular carcinoma	355 (97.3%)
RX	20 (5.5%)	Fibrolamellar carcinoma	3 (0.8%)
Not applicable	7 (1.9%)	Hepatocholangiocarcinoma	7 (1.9%)

**Fig. 4:**
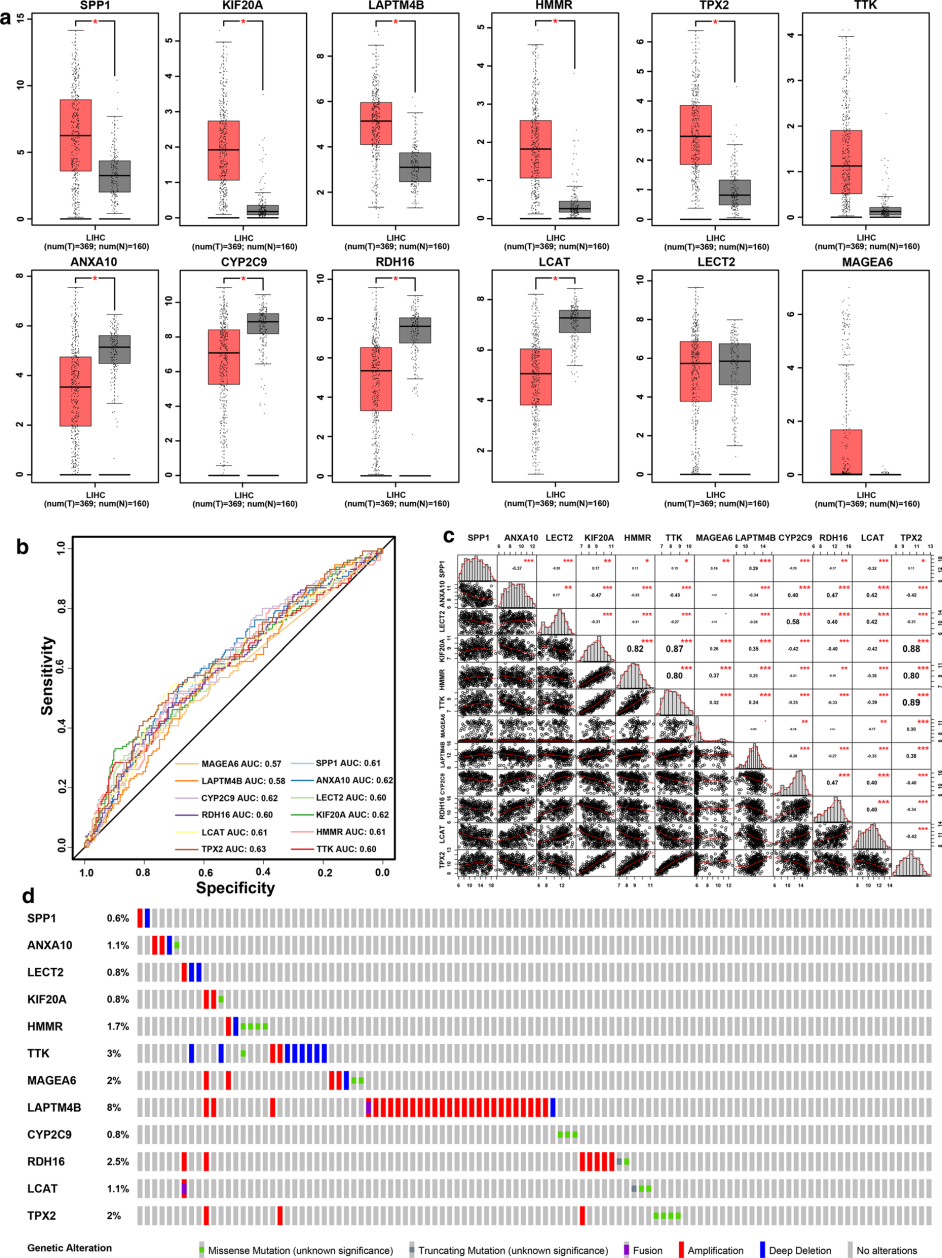
Validation of expression and alteration of the twelve genes in hepatocellular carcinoma. **a** Expression of the twelve genes were validated in 369 HCC tissues (red) and 160 normal tissues (gray) with GEPIA. **b** ROC analysis revealed a well diagnostic performance of the twelve genes with HCC. **c** Correlation analysis among the twelve genes. **d** Genetic alterations of the twelve genes in HCC. Data were obtained from the cBioportal

To validate different expressions of the twelve genes between tumor and non-tumor tissue, 369 HCC tissues and 160 normal tissues were compared using Gene Expression Profiling Interactive Analysis (GEPIA, http://gepia.cancer-pku.cn/). The mRNA expression levels of SPP1, KIF20A, HMMR, LAPTM4B and TPX2 were significantly increased in HCC tissues, while the levels of ANX10, CYP2C9, LCAT and RDH16 were significantly decreased (Fig. 4a). ROC analysis showed well prognostic performance of the twelve-gene signature (Fig. 4b). The Kaplan–Meier survival curves revealed that the upregulated gene with lower expression levels have better survival period, while the downregulated genes were positive correlation with survival period (*P* value < 0.05; Additional file 8: Figure S5). Besides, correlation analysis indicated that TPX2 and TTK had a strong positive correlation (r = 0.89, *P* < 0.001), and that KIF20A and ANXA10 had a moderate negative correlation (r = − 0.47, *P* < 0.001) (Fig. 4c; Additional file 9: Table S4). Of the 365 TCGA-LIHC patients included in the mutation analysis, 88 (23.6%) presented with alterations in the twelve genes. Amplification was the most common type of mutation among the upregulated genes especially LAPTM4B (Fig. 4d). Typical IHC of twelve genes (except LCAT and MAGEA6, not included in the database) in tumor and normal liver tissues are shown in Additional file 10: Figure S6.

**Fig. 5:**
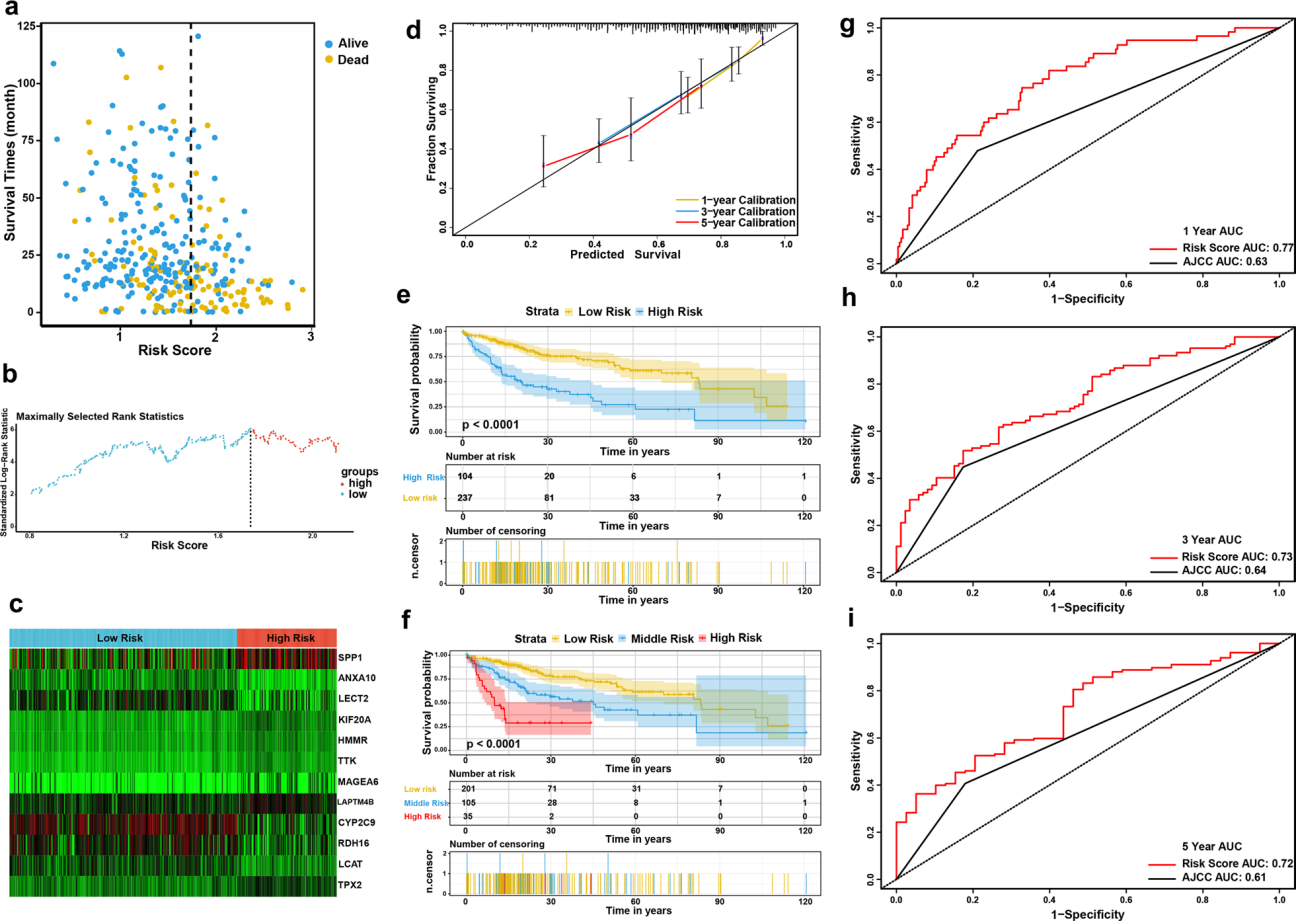
Evaluation of the prognostic performance of the twelve-gene signature in TCGA-LIHC dataset. **a** Distribution of the risk score and survival data. Alive cases showed in blue, dead cases showed in yellow. **b** Distribution of the risk score. **c** Heat map of the twelve gene expression in the TCGA-LIHC dataset. **d** Calibration plot of the twelve-gene signature for predicting the probability of survival at 1-, 3-, and 5-years. **e**, **f** Kaplan–Meier survival curves of the risk score model. **g**–**i** Time-dependent ROC curve of risk score model for 1-, 3-, and 5-year overall survival predictions in compare with AJCC stage

Then the risk score was calculated for each patient incorporating the corresponding coefficients as follows: [(− 0.0627) × Expression value of ANXA10] + [(− 0.03552) × Expression value of CYP2C9] + [(− 0.02365) × Expression value of LCAT] + [(− 0.02237) × Expression value of LECT2] + [(− 0.01796) × Expression value of RDH16] + [(0.0015) × Expression value of LAPTM4B] + [(0.0386) × Expression value of KIF20A] + [(0.05124) × Expression value of SPP1] + [(0.05284) × Expression value of MAGEA6] + [(0.05377) × Expression value of TPX2] + [(0.0565) × Expression value of HMMR] + [(0.09788) × Expression value of TTK]. Subsequently, the included 341 TCGA-LIHC patients were stratified into two (cutoff value = 1.74) or three (cutoff values = 1.58 and 2.11) groups. The Kaplan–Meier survival curves revealed significantly favorable OS in the groups with lower risk score (*P* < 0.0001) (Fig. 5e, f). Time-dependent ROC curve and C-index analyses were applied to evaluate the prognostic values of the twelve-gene risk scores and were then compared with those of the AJCC stage (Fig. 5g–i). The AUCs for the 1-, 3-, and 5-year OS predictions for the risk score were 0.77, 0.73, and 0.72, respectively. The AUCs for the 1-, 3-, and 5-year OS predictions for the AJCC stage were 0.63, 0.64, and 0.61, respectively. The C-index of the risk score was 0.653 (95% CI 0.606–0.700), while that of the AJCC stage was 0.591 (95% CI 0.544–0.638).

### External validation of the prognostic performance of the twelve-gene signature

**Fig. 6:**
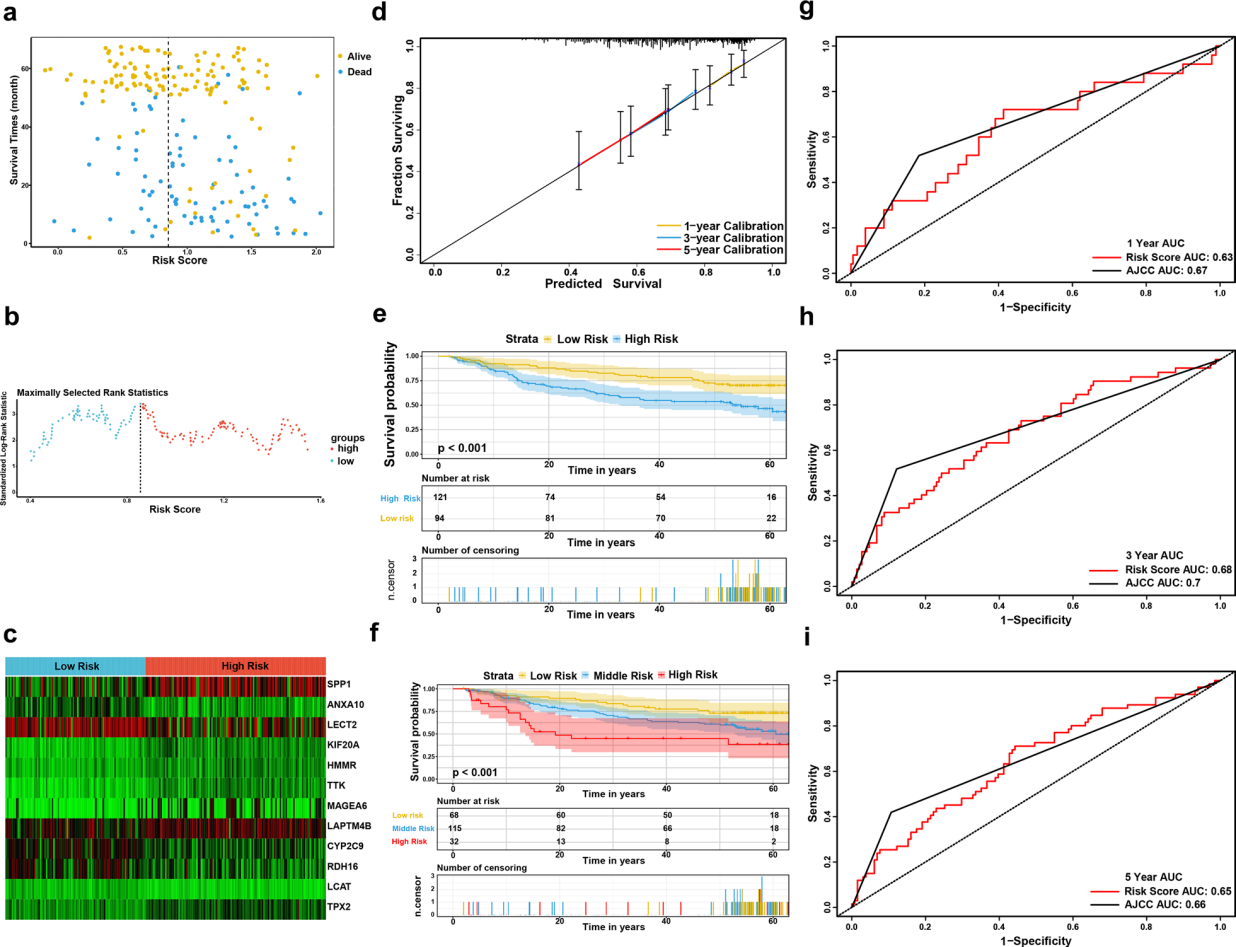
External validation of the prognostic performance of the twelve-gene signature in GSE14520 dataset. **a** Distribution of the risk score and survival data. Alive cases showed in yellow, dead cases showed in blue. **b** Distribution of the risk score. **c** Heat map of the twelve gene expression in the GSE14520 dataset. **d** Calibration plot of the twelve-gene signature for predicting the probability of survival at 1-, 3-, and 5-years. **e**, **f** Kaplan–Meier survival curves of the risk score model. **g**–**i** Time-dependent ROC curve of risk score model for 1-, 3-, and 5-year overall survival predictions in compare with AJCC stage

To validate the classification performance of the twelve-gene prognostic signature with different data platforms, we used the GSE14520 cohort, which included 216 HCC patients, as an external dataset. Similarly, the patients each received exclusive risk score and were stratified into two or three groups based on cutoff value calculated by X-Tile. The Kaplan–Meier survival curves showed significant difference in the OS among the groups. Low-risk group had obviously better outcomes than high-risk or middle-risk groups (Fig. 6e, f). Moreover, the AUCs for the 1-, 3-, and 5-year OS predictions for the risk score were 0.63, 0.68 and 0.66, respectively, which show comparable prognostic power with the AJCC stage (Fig. 6g–i). The C-index of the risk score was 0.614 (95% CI 0.549–0.679), while that of the AJCC stage was 0.622 (95% CI 0.573–0.671). External validation suggested that the twelve-gene signature continued to perform well at predicting OS in HCC patients.

### Generation and validation of the diagnostic performance of the twelve-gene signature

**Fig. 7:**
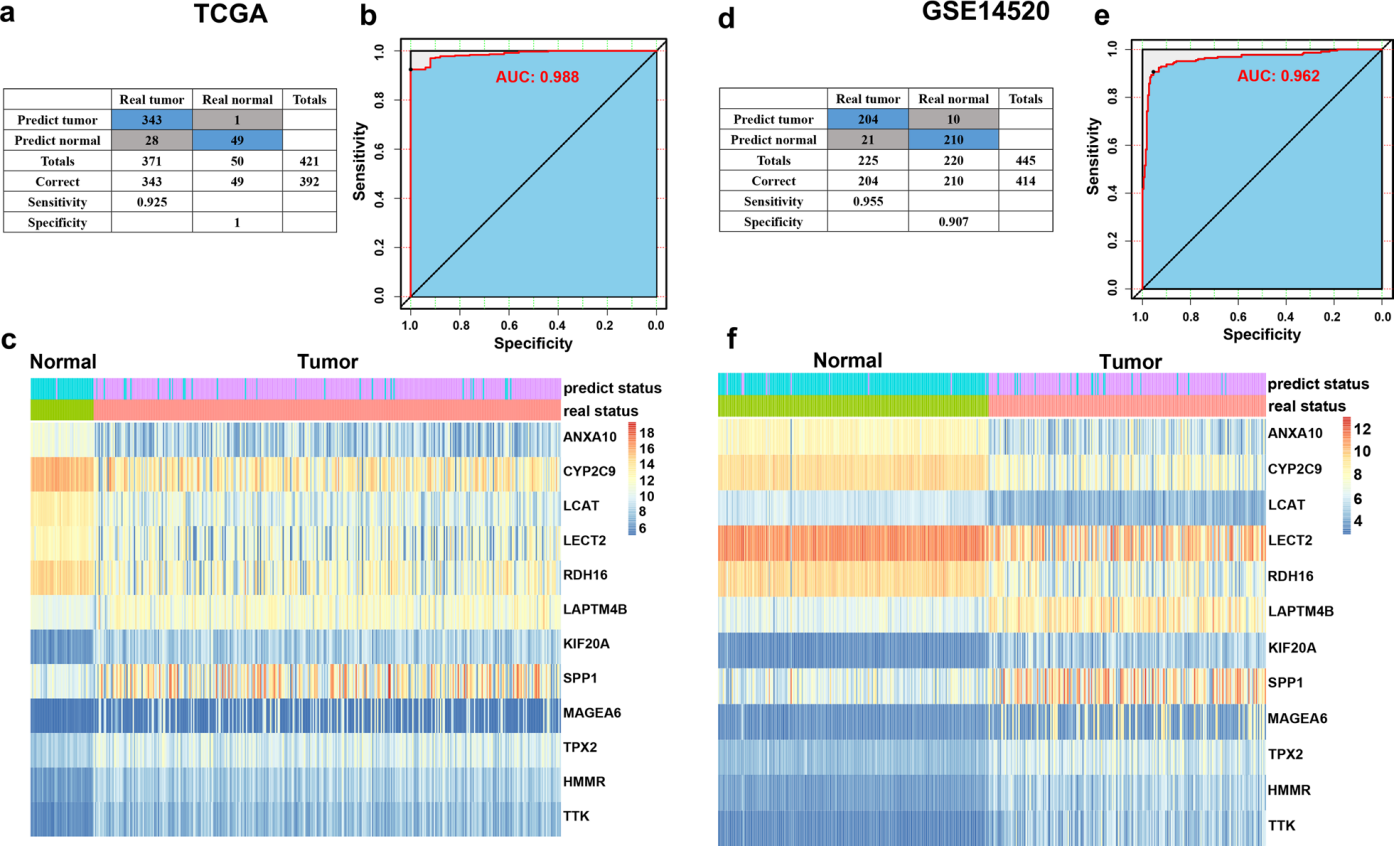
The diagnostic performance of the twelve-gene signature in distinguishing HCC from normal samples. Crosstab of diagnostic prediction model for training (**a**) and validation (**d**) dataset. ROC curves of the diagnostic prediction models with the twelve genes for training (**b**) and validation (**e**) datasets. **c**, **f** Unsupervised hierarchical clustering of twelve genes in the diagnostic prediction model for training (**e**) and validation (**f**) datasets

As for training datasets (TCGA-LIHC) of 371 HCC samples and 50 normal samples, we established a diagnostic model with twelve genes by using the logistic regression analysis to distinguish HCC from normal tissue. Exclusive diagnostic score was calculated by corresponding coefficients as follows: 1.5343 + [(− 0.0198) × Expression value of ANXA10] + [(− 0.01206) × Expression value of CYP2C9] + [(− 0.04958) × Expression value of LCAT] + [(0.00453) × Expression value of LECT2] + [(− 0.01166) × Expression value of RDH16] + [(0.00351) × Expression value of LAPTM4B] + [(0.06177) × Expression value of KIF20A] + [(− 0.00863) × Expression value of SPP1] + [(0.00155) × Expression value of MAGEA6] + [(− 0.14711) × Expression value of TPX2] + [(0.12204) × Expression value of HMMR] + [(0.03522) × Expression value of TTK]. The model showed a strong diagnosis performance of 92.5% sensitivity and 100% specificity to distinguish HCC from normal samples in TCGA datasets (AUC = 0.988) (Fig. 7a, b), while 95.5% sensitivity and 90.7% specificity in the GSE14520 datasets (AUC = 0.962) of 225 HCC samples and 220 normal samples (Fig. 7d, e). Unsupervised hierarchical clustering of twelve genes could also differentiate HCC from normal tissue with high sensitivity and specificity (Fig. 7c, f).

### Correlation with clinicopathological characteristics and prognostic factor

Among the 233 patients included in TCGA-LIHC cohort with complete clinical information, a higher risk score was found to be significantly correlated with female sex, advanced tumor grade, vascular invasion and higher AFP (Table 3). Moreover, the univariable and multivariable Cox regression analyses indicated that the risk score and AJCC stage were both independent prognostic factors for OS (Table 4).

**Table 3:** Correlation of clinicopathologic characteristics and the twelve-gene signature in HCC

Characteristics	TCGA-LIHC			GSE14520		
	Low risk N = 176	High risk N = 57	*P*	Low risk N = 94	High risk N = 121	*P*
Follow-up time (mouths)	31.49 ± 25.24	21.85 ± 18.99	0.009	47.51 ± 18.72	34.36 ± 22.00	0.000
Risk score	1.23 ± 0.35	2.05 ± 0.25	0.000	0.54 ± 0.22	1.29 ± 0.29	0.000
Age (years)			0.565			0.282
≤ 60	88 (50.0%)	26 (45.6%)		73 (77.7%)	101 (83.5%)	
> 60	88 (50.0%)	31 (54.4%)		21 (22.3%)	20 (16.5%)	
Sex			0.008			0.595
Female	50 (28.4%)	27 (47.4%)		14 (14.9%)	15 (12.4%)	
Male	126 (71.6%)	30 (52.6%)		80 (85.1%)	106 (87.6%)	
BMI (kg/m^2^)			0.664			
< 25	93 (52.8%)	32 (56.1%)		–	–	
≥ 25	83 (47.2%)	25 (43.9%)		–	–	
G stage			0.000			
G1 + G2	111 (63.1%)	17 (29.8%)		–	–	
G3 + G4	65 (36.9%)	40 (70.2%)		–	–	
Residual tumor			0.905			
R0	166 (94.3%)	54 (94.7%)		–	–	
Non-R0	10 (5.7%)	3 (5.3%)		–	–	
AJCC stage			0.089			0.006
I + II	150 (85.2%)	43 (75.4%)		81 (86.2%)	85 (70.2%)	
III + IV	26 (14.8%)	14 (24.6%)		13 (13.8%)	36 (29.8%)	
Vascular invasion			0.000			
No	129 (73.3%)	23 (40.4%)		–	–	
Yes	47 (26.47%)	34 (59.6%)		–	–	
AFP (ng/ml)			0.002			0.001
< 300	144 (81.8%)	35 (61.4%)		64 (68.1%)	54 (44.6%)	
≥ 300	32 (18.2%)	22 (38.6%)		30 (31.9%)	67 (55.4%)	
Multinodular						0.214
No	–	–		78 (83.0%)	92 (76.0%)	
Yes	–	–		16 (17.0%)	29 (24.0%)	
Cirrhosis						0.575
No	–	–		9 (9.6%)	9 (7.4%)	
Yes	–	–		85 (90.4%)	112 (92.6%)

**Table 4:** Univariate and multivariate Cox regression analysis of TCGA-LIHC patients

Characteristics	Univariate Cox		Multivariate Cox	
	HR (95%CI)	*P*	HR (95%CI)	*P*
Risk score	2.948 (1.752–4.961)	0.000	2.349 (1.289–4.279)	0.005
Age (years)				
< 60	1		1	
≥ 60	1.535 (0.923–2.552)	0.099	1.593 (0.935–2.714)	0.086
Sex				
Female	1			
Male	0.673 (0.405–1.119)	0.127	1.023 (0.588–1.779)	0.936
BMI (kg/m^2^)				
< 25	1		–	–
≥ 25	1.214 (0.738–1.999)	0.445	–	–
G stage				
G1 + G2	1		1	
G3 + G4	1.536 (0.930–2.536)	0.094	1.364 (0.791–2.352)	0.265
Residual tumor				
R0	1		–	–
Non-R0	1.328 (0.413–4.278)	0.634	–	–
AJCC stage				
I + II	1		1	
III + IV	2.271 (1.321–3.904)	0.003	2.219 (1.215–3.734)	0.008
Vascular invasion				
No	1		1	
Yes	1.760 (1.048–2.958)	0.033	1.266 (0.730–2.196)	0.401
AFP (ng/ml)				
< 300	1		–	–
≥ 300	1.171 (0.668–2.054)	0.581	–	–

### Building and validating a predictive nomogram from the TCGA‐LIHC cohort

**Fig. 8:**
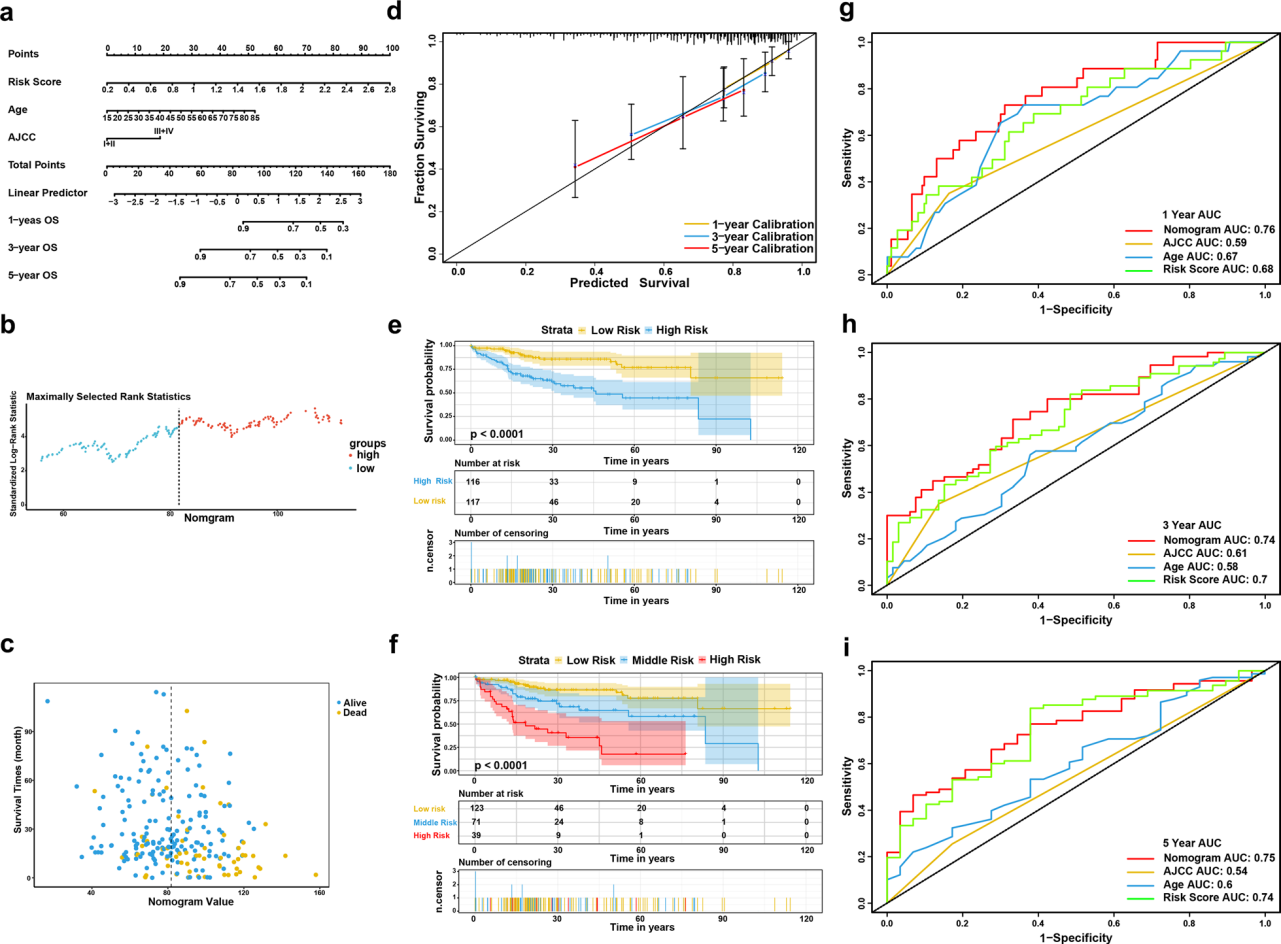
Validation of the nomogram in predicting overall survival of the TCGA-LIHC cohort. **a** A prognostic nomogram predicting 1-, 3-, and 5-year overall survival of HCC. **b** Distribution of the nomogram score. **c** Distribution of the nomogram score and survival data. Alive cases showed in blue; dead cases showed in yellow. **d** Calibration plot of the nomogram for predicting the probability of survival at 1-, 3-, and 5-years. **e**, **f** Kaplan–Meier survival curves of the nomogram. **g**–**i** Time-dependent ROC curve of the nomogram for 1-, 3-, and 5-year overall survival predictions in compare with AJCC stage

The 233 TCGA-LIHC patients with complete clinical information were adopted to build a prognostic nomogram. Risk score, age and AJCC stage were used as parameters in the nomogram (Fig. 8a). The AUCs of the 1-, 3-, and 5-year OS predictions for the nomogram were 0.76, 0.74, and 0.75, respectively (Fig. 8g–i). The C-index of the nomogram was 0.711 (95% CI 0.642–0.78), while that for the AJCC stage was 0.567 (95% CI 0.508–0.626). Thus, the nomogram was superior to the risk score or AJCC stage in predicting OS of HCC. The patients were stratified into two or three groups based on median or cutoff values generated by X-Tile according to the scoring of the nomogram. The Kaplan–Meier curves showed significant difference in the OS among groups (Fig. 8e, f). Those with lower scores experienced significantly better survival period (*P* < 0.0001). Calibration plots showed that the nomogram performed well at predicting OS in HCC patients (Fig. 8d).

### Validation of the DNA methylation pattern of twelve-gene signature

**Fig. 9:**
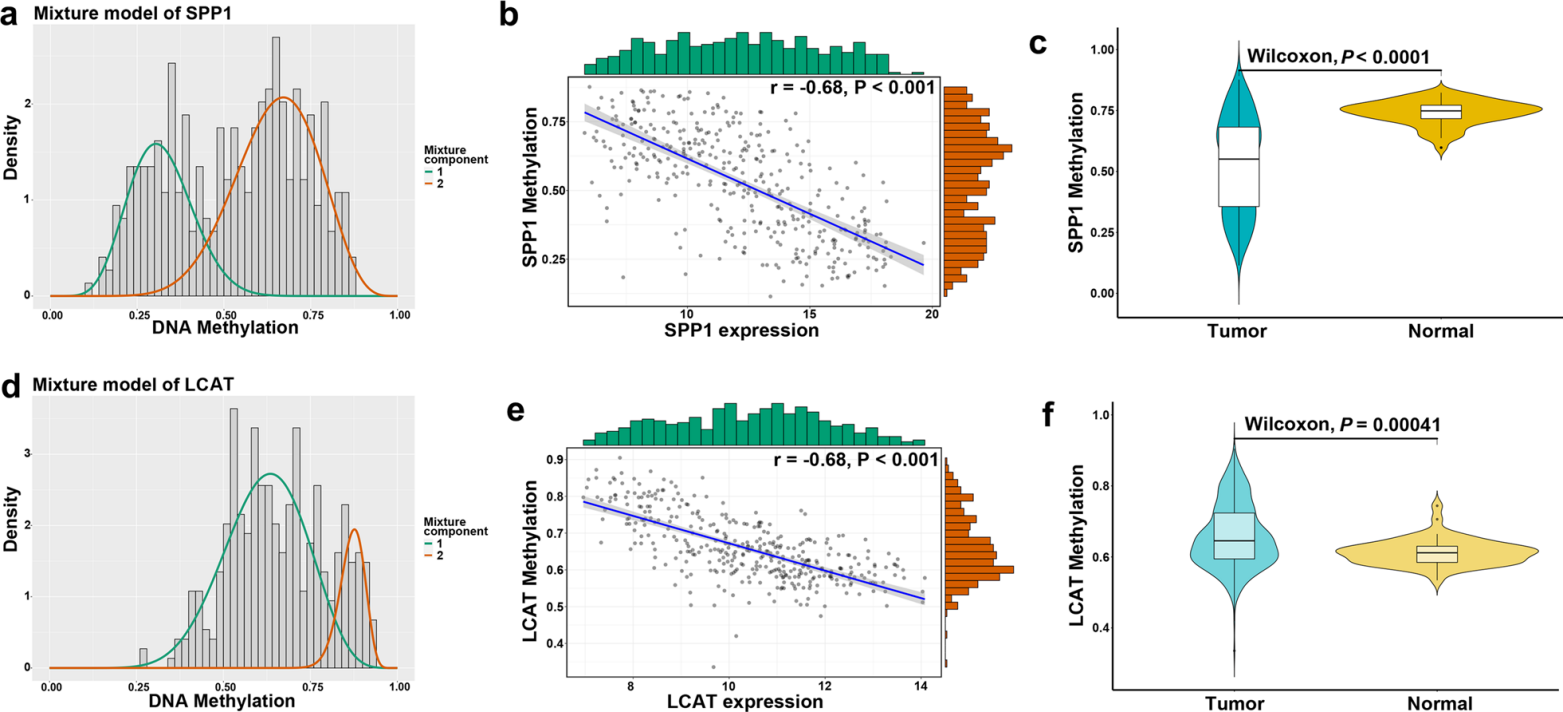
The DNA methylation pattern of twelve-gene signature. **a**, **d** Mixture models for SPP1 and LCAT. The horizontal black bar indicates the distribution of methylation values in normal samples. The histogram illustrates the distribution of methylation in tumor samples (signified as beta values, where higher beta values denote greater methylation). **b**, **e** Regression analysis between gene expression and DNA methylation of SPP1 and LCAT. **c**, **f** Violin plots of the DNA methylation status of SPP1 and LCAT

Based on the DNA methylation data and the paired gene expression data of twelve genes in 371 HCC tissues, functional DNA methylation analyses showed that six genes, including SPP1, RDH16, LAPTM4B, LCAT, CYP2C9 and LECT2, had a significantly strong negative correlation between with gene expression and DNA methylation, and four genes (HMMR, KIF20A, TPX2 and TTK) showed moderate or weak correlation (Fig. 9b, e, Additional file 11: Figure S7), while the methylation data involving ANXA10 and MAGEA6 were lacked. Besides, the beta mixture model had identified SPP1 and LCAT as the DNA methylation-driven genes, which the gene expression value was significantly affected by DNA methylation events. A significantly low DNA methylation were noted for SPP1 relative to high expression levels in tumor tissues, while high DNA methylation and low expression for LCAT (*P* < 0.0001) (Fig. 9).

## Discussion

For decades, many studies have strived to elucidate the pathogenesis and epidemiology of HCC. Although great improvement on surgical and medical therapy has been made, the prognosis of HCC remains poor, which has resulted in a heavy disease burden on a global scale. Lacking of efficient detection methods on the early stage attributes to the progression of the disease. Moreover, HCC is a highly heterogeneous disease in that survival times vary significantly among patients with similar TNM stages. Consequently, screening an efficient prognostic marker that dynamically reflect the biological progression of the tumor would be important for individualized prevention and treatment of HCC.

The present study aimed to identify an effective prognostic marker to stratify HCC patients and predict the outcome of HCC. In our current study, a total of 175 overlapping DEGs between HCC tissues and non-tumor tissues were identified from six GEO datasets after integrating by the RRA method. Eighty prognosis-related genes were sifted out from TCGA datasets by using univariable Cox regression methods, which were then subjected to Lasso regression with tenfold cross-validation. Finally, a novel twelve-gene signature was determined. The risk score of each case was calculated based on the above model, which stratified HCC patients into high- or low-risk groups. The twelve-gene signature was an independent prognostic factor of HCC according to the Kaplan–Meier survival curves analysis and ROC analysis and verified by TCGA datasets and GSE14520 dataset, and it was comparable or superior to AJCC stage at predicting 1-, 3-, and 5-year OS. In addition, the twelve-gene signature was independent with other clinical factors and performed better in predicting OS in combination with age and AJCC stage in a nomogram.

To date, many studies have explored the prognostic role of gene signatures in predicting the outcome of HCC. The effect of clinical practice based on gene expression profiles has shown that gene signatures might be a promising high-throughput molecular identification method [22–24]. In that regard, Liu et al. [25] identified a four‐gene metabolic signature and Xiang et al. [26] identified seven-senescence-associated gene signature for predicting OS in HCC. Good predictive performance was obtained in their respective studies. However, our 12-gene signature had a higher 1-year, 3-year, and 5-year AUC than the above two gene signatures, which makes it more conducive to clinical application in several clinical important settings (Additional file 12: Figure S8). Sometimes, patients with similar clinicopathological features, such as AJCC stage, may have distinct outcomes, which might due to the heterogeneity of the different epigenetic and genetic backgrounds in tumor subtypes. Therefore, the combined use of the twelve gene prognostic signature and AJCC stage might be benefit to identify high-risk patients who should receive more medical supports.

The initiation and progression of HCC is a complex and multistage process regulated by both genetic and epigenetic alterations. DNA methylation, as a central epigenetic modification, would facilitate tumor development if it was dysfunctional, such as hypomethylation of oncogenes and hypermethylation of tumor suppressor [27, 28]. In present study, most of the prognostic twelve genes were negatively correlated with DNA methylation in HCC tissues. As the DNA methylation-driven genes identified by the beta mixture model, SPP1 was hypomethylated and expressed at a higher level in HCC than in normal tissues, while LCAT was hypermethylated and low expression. Those results could be a validation and extension of the research initiated by Long et al. [28]. They found that the models consisting of SPP1 and LCAT were good at predicting HCC diagnosis, prognosis and recurrence. However, the marker involving in more pathogenic pathways not just DNA methylation might guide better clinical due to the complex biological process related with HCC pathogenesis.

The five of twelve-gene signature were downregulated in HCC tissues and identified as protective genes, including ANXA10, CYP2C9, LCAT, LECT2 and RDH16. ANXA10 is one of 13 annexins members of superfamily of calcium-dependent phospholipid-binding proteins [29]. Downregulation of ANXA10 is associated with worse pathological stage and poor prognosis in liver cancer [30, 31]. Previous study has identified it as a prognostic biomarker of perihilar and distal cholangiocarcinoma but not intrahepatic cholangiocarcinoma, the gene promotes extrahepatic cholangiocarcinoma metastasis by facilitating the epithelial-mesenchymal transition via the PLA2G4A/PGE2/STAT3 pathway [32]. CYP2C9, a gene involved in drug absorption, distribution, metabolism and excretion, was downregulated in HCC tissue in part due to the de-differentiation of cancer cells [33]. It is associated with loss of liver-specific functions. LACT plays an important role in many cancers and was hypermethylated and downregulated in HCC compared with non-tumor tissue [34]. LECT2, a chemokine-like chemotactic factor, plays a protective anti-inflammatory role in the liver with β-catenin-induced tumorigenesis, and downregulated LECT2 expression results in the presence of inflammatory infiltrates, tumor progression and metastatic disease [35]. This protective effect is mainly targeted both tumoral hepatocytes and the hepatic immune microenvironment by inhibiting the Wnt pathway [36]. RDH16 is strongly express in normal liver tissue and is involved in retinoic acid metabolism, cellular proliferation, differentiation, and apoptosis [37]. The level of RDH16 expression is inversely associated with tumor size, microsatellite formation, thrombus and OS in HCC patients [38].

The seven of the twelve genes were upregulated in HCC tissue and were associated with poor survival, including SPP1, KIF20A, HMMR, TTK, MAGEA6, LAPTM4B and TPX2. The SPP1 gene, located on chromosome 4 in locus 4q13.22 [39], encodes a secreted chemokine-like glycophospho protein, named as the glycoprotein osteopontin. The biological functions of osteopontin are diverse [40]. It is overexpressed during the development and progression of different cancers, and has been suggested as a potential prognostic biomarker and therapeutic target [41]. KIF20A accumulates in the nucleus during the G2 phase of the cell cycle [42], and contributed to cellular proliferation, apoptosis and metastasis by regulating various signaling pathways, such as the E2F-retinoblastoma protein-p16 pathway and the PI3K/Akt signaling pathways. Intracellular HMMR participates in mitotic spindle pole formation and cytokinesis [43]. Overexpression of HMMR is associated with cancer growth, migration, and poor prognosis in various cancers, including HCC, via its stimulation of the hyaluronan-HMMR signaling cascade in addition to its oncogenic activities as noted above [44]. TTK is essential for the mitotic checkpoint and improper chromosome attachments [45]. Elevated TTK level facilitates chromosome instability and aneuploidy, thus contributing to cancer cell proliferation and invasion [46]. The essential role of TTK in HCC carcinogenesis by promoting cell survival and invasion via activation of Akt/mTOR and MDM2/p53 signaling pathways, and the regulation of miR-21 via TGF-β [47]. MAGEA6 is generally expressed in the male testis, but is re-activated in many tumor cells. MAGEA6 and TRIM28 was combined to form a cancer-specific ubiquitin ligase that inhibited AMPK signaling pathway and induced the stemness maintenance and self‐renewal of HCC stem cells [48]. LAPTM4B was originally found to be overexpressed in HCC tissue, and it has inverse correlation with HCC differentiation. Evidences suggested that the overexpression of LAPTM4B may promote malignant transformation and enhance the metastasis and recurrence of HCC via activating AKT signaling pathway and some proto-oncogenes such as c-myc, c-jun and c-fos [49, 50]. TPX2 is a nuclear proliferation microtubule-associated protein that can regulate spindle formation and stabilize spindle microtubules by promoting chromatin microtubule nucleation. Targeted silencing of TPX2 inhibited cell viability, disturbed cell cycle process and induced apoptosis of HCC cells by inhibiting the PI3K/AKT signaling transduction pathway.

Although we have identified prognosis-related gene signature which showed potentially substantial clinical significance, the present study had certain limitations. Considering the great heterogeneity of HCC, some important candidate genes affecting tumor prognosis might have been excluded before constructing the prognostic model, which could have decreased the performance of the model in the TCGA cohort. Moreover, the mechanisms of post‐curative recurrence and metastasis (the major obstacles of HCC survival) might also help explain the relative low diagnostic performance, but the clinical follow-up information, including post‐curative recurrence and metastasis data, were lacking in our included samples. In addition, the twelve-gene prognostic signature needs to be investigated by experimental verification, and evaluated for clinical application using multicenter randomized controlled studies.

## Conclusions

In conclusion, we identified a prognostic twelve gene signature via comprehensive bioinformatics analysis with TCGA and GEO liver cancer cohorts. It could effectively stratify HCC patients into high- and low-risk group and independently predict the survival of HCC patients. Our finding indicated that the twelve gene signature may help facilitate personalized cancer management in the clinical setting. We therefore recommend using this classifier as a molecular diagnostic test to evaluate the prognostic risk in HCC patients.

## Supplementary information

**Additional file 1: Figure S1.** Normalization and distribution of mRNA expression in six GEO datasets. (A) GSE45267 dataset; (B) GSE65372 dataset; (C) GSE51401 dataset; (D) GSE121248 dataset; (E, F) GSE14520 dataset.

**Additional file 2: Table S1.** 175 DEGs identified by integrated analysis using robust rank aggregation (RRA) method with adjPvalue < 0.05.

**Additional file 3: Figure S2.** Functional analysis. (A) KEGG analysis of the DEGs; (B) the top 25 hub genes analyzed by the PPI network.

**Additional file 4: Table S2.** KEGG pathway analysis of the DEGs.

**Additional file 5: Figure S3.** PPI network analysis of the DEGs. (A) Clustering module 1 with a score of 70.421 and its top 20 most enriched biological processes. (B) Clustering module 2 with a score of 11.12 and its top 20 most enriched biological processes. (C) Clustering module 3 with a score of 6.121 and its top 20 most enriched biological processes.

**Additional file 6: Figure S4.** LASSO coefficient profiles (A) and LASSO deviance profiles (B).

**Additional file 7: Table S3.** DEGs associated with overall survival using univariate Cox regression model with P < 0.01.

**Additional file 8: Figure S5.** Kaplan–Meier survival curves of the twelve genes.

**Additional file 9. Table S4.** Pearson correlation analyses among twelve-gene signatures in 341 TCGA LIHC Samples.

**Additional file 10: Figure S6**. Typical IHC of twelve genes (except LCAT and MAGEA6, not included in the database) in tumor and normal liver tissues.

**Additional file 11: Figure S7.** Regression analysis between eight gene expression and DNA methylation.

**Additional file 12: Figure** **S8.** Time-dependent ROC curve of the risk score for 1-, 3-, and 5-year overall survival predictions in compare with other studies.

## Data Availability

The data included in this study originate from the public free-charged database including The Gene Expression Omnibus (https://www.ncbi.nlm.nih.gov/geo/) and The Cancer Genome Atlas (https://portal.gdc.cancer.gov/).

## Abbreviations

HCC: Hepatocellular carcinoma
TCGA: The Cancer Genome Atlas
GEO: Gene Expression Omnibus
LIHC: Liver hepatocellular carcinoma
DEGs: Differentially expressed genes
ROC: Receiver operating characteristic
AJCC: American Joint Committee on Cancer
KEGG: Kyoto Encyclopedia of Genes and Genomes
OS: Overall survival
GO: Gene Ontology
logFC: Log2-based fold change
RRA: Robust rank aggregation
AUC: Area under curve
HR: Hazard ratio
CI: Confidence interval
SPP1: Secreted phosphoprotein 1
ANXA10: Annexin10
LECT2: Leukocyte cell-derived chemotaxin 2
KIF20A: Kinesin family member 20A
HMMR: Hyaluronan mediated motility receptor
MAGEA6: Melanoma antigen A6
LAPTM4B: Lysosomal protein transmembrane 4 beta
CYP2C9: Cytochrome P450 family 2 subfamily C member 9
RDH16: Retinol dehydrogenase 16
LCAT: Lecithin-cholesterol acyltransferase
TPX2: Targeting protein for xenopus kinesin-like protein 2

## References

[1] Bray F, Ferlay J, Soerjomataram I, Siegel RL, Torre LA, Jemal A (2018). Global cancer statistics 2018: GLOBOCAN estimates of incidence and mortality worldwide for 36 cancers in 185 countries. CA Cancer J Clin.

[2] Kulik L, El-Serag HB (2019). Epidemiology and Management of Hepatocellular Carcinoma. Gastroenterology.

[3] Llovet JM, Villanueva A, Lachenmayer A, Finn RS (2015). Advances in targeted therapies for hepatocellular carcinoma in the genomic era. Nat Rev Clin Oncol.

[4] Hiraoka A, Kumada T, Tsuji K, Takaguchi K, Itobayashi E, Kariyama K, Ochi H, Tajiri K, Hirooka M, Shimada N (2019). Validation of modified ALBI grade for more detailed assessment of hepatic function in hepatocellular carcinoma patients: a multicenter analysis. Liver Cancer.

[5] Hiraoka A, Michitaka K, Kumada T, Izumi N, Kadoya M, Kokudo N, Kubo S, Matsuyama Y, Nakashima O, Sakamoto M (2019). Prediction of prognosis of intermediate-stage HCC patients: validation of the tumor marker score in a nationwide database in Japan. Liver Cancer.

[6] Du J, Zhao Z, Zhao H, Liu D, Liu H, Chen J, Cheng B, Zhai X, Yin Z, Zhang Y (2019). Sec62 promotes early recurrence of hepatocellular carcinoma through activating integrinalpha/CAV1 signalling. Ooncogenesis.

[7] Wen LZ, Ding K, Wang ZR, Ding CH, Lei SJ, Liu JP, Yin C, Hu PF, Ding J, Chen WS (2018). SHP-1 acts as a tumor suppressor in hepatocarcinogenesis and HCC progression. Cancer Res.

[8] Shen J, Li P, Shao X, Yang Y, Liu X, Feng M, Yu Q, Hu R, Wang Z (2018). The E3 ligase RING1 targets p53 for degradation and promotes cancer cell proliferation and survival. Cancer Res.

[9] Wang LL, Jin XH, Cai MY, Li HG, Xie D (2017). AGBL2 promotes cancer cell growth through IRGM-regulated autophagy and enhanced Aurora A activity in hepatocellular carcinoma. Cancer Lett.

[10] Liu G, Zeng H, Zhang C, Xu J (2019). Identification of a six-gene signature predicting overall survival for hepatocellular carcinoma. Cancer Cell Int.

[11] Wang Z, Teng D, Li Y, Hu Z, Liu L, Zheng H (2018). A six-gene-based prognostic signature for hepatocellular carcinoma overall survival prediction. Life Sci.

[12] Long J, Zhang L, Wan X, Lin J, Bai Y, Xu W, Xiong J, Zhao H (2018). A four-gene-based prognostic model predicts overall survival in patients with hepatocellular carcinoma. J Cell Mol Med.

[13] Gao J, Aksoy BA, Dogrusoz U, Dresdner G, Gross B, Sumer SO, Sun Y, Jacobsen A, Sinha R, Larsson E (2013). Integrative analysis of complex cancer genomics and clinical profiles using the cBioPortal. Sci Signal.

[14] Ritchie ME, Phipson B, Wu D, Hu Y, Law CW, Shi W, Smyth GK (2015). limma powers differential expression analyses for RNA-sequencing and microarray studies. Nucleic Acids Res.

[15] Tibshirani R (1997). The lasso method for variable selection in the Cox model. Stat Med.

[16] Friedman J, Hastie T, Tibshirani R (2010). Regularization Paths for Generalized Linear Models via Coordinate Descent. J Stat Softw.

[17] Robin X, Turck N, Hainard A, Tiberti N, Lisacek F, Sanchez JC, Muller M (2011). pROC: an open-source package for R and S + to analyze and compare ROC curves. BMC Bioinform.

[18] Heagerty PJ, Lumley T, Pepe MS (2000). Time-dependent ROC curves for censored survival data and a diagnostic marker. Biometrics.

[19] Iasonos A, Schrag D, Raj GV, Panageas KS (2008). How to build and interpret a nomogram for cancer prognosis. J Clin Oncol.

[20] Cedoz PL, Prunello M, Brennan K, Gevaert O (2018). MethylMix 2.0: an R package for identifying DNA methylation genes. Bioinformatics.

[21] Kolde R: pheatmap: Pretty Heatmaps. 2015.

[22] Bhutiani N, Egger ME, Ajkay N, Scoggins CR, Martin RN, McMasters KM (2018). Multigene signature panels and breast cancer therapy: patterns of use and impact on clinical decision Making. J Am Coll Surg.

[23] Wang SY, Dang W, Richman I, Mougalian SS, Evans SB, Gross CP (2018). Cost-effectiveness analyses of the 21-gene assay in breast cancer: systematic review and critical appraisal. J Clin Oncol.

[24] Kopetz S, Tabernero J, Rosenberg R, Jiang ZQ, Moreno V, Bachleitner-Hofmann T, Lanza G, Stork-Sloots L, Maru D, Simon I (2015). Genomic classifier ColoPrint predicts recurrence in stage II colorectal cancer patients more accurately than clinical factors. Oncologist.

[25] Liu GI, Xie WU, Zhang CU, Xu JE (2019). Identification of a four gene metabolic signature predicting overall survival for hepatocellular carcinoma. J Cell Physiol.

[26] Xiang XH, Yang L, Zhang X, Ma XH, Miao RC, Gu JX, Fu YN, Yao Q, Zhang JY, Liu C (2019). Seven-senescence-associated gene signature predicts overall survival for Asian patients with hepatocellular carcinoma. World J Gastroenterol.

[27] Edwards JR, Yarychkivska O, Boulard M, Bestor TH (2017). DNA methylation and DNA methyltransferases. Epigenet Chromatin.

[28] Long J, Chen P, Lin J, Bai Y, Yang X, Bian J, Lin Y, Wang D, Yang X, Zheng Y (2019). DNA methylation-driven genes for constructing diagnostic, prognostic, and recurrence models for hepatocellular carcinoma. Theranostics.

[29] Enrich C, Rentero C, Grewal T (2017). Annexin A6 in the liver: from the endocytic compartment to cellular physiology. Biochim Biophys Acta Mol Cell Res.

[30] Liu SH, Lin CY, Peng SY, Jeng YM, Pan HW, Lai PL, Liu CL, Hsu HC (2002). Down-regulation of annexin A10 in hepatocellular carcinoma is associated with vascular invasion, early recurrence, and poor prognosis in synergy with p53 mutation. Am J Pathol.

[31] Zhuang C, Wang P, Sun T, Zheng L, Ming L (2019). Expression levels and prognostic values of annexins in liver cancer. Oncol Lett.

[32] Sun R, Liu Z, Qiu B, Chen T, Li Z, Zhang X, Xu Y, Zhang Z (2019). Annexin10 promotes extrahepatic cholangiocarcinoma metastasis by facilitating EMT via PLA2G4A/PGE2/STAT3 pathway. EBiomedicine.

[33] Hu DG, Marri S, McKinnon RA, Mackenzie PI, Meech R (2019). Deregulation of the genes that are involved in drug absorption, distribution, metabolism, and excretion in hepatocellular carcinoma. J Pharmacol Exp Ther.

[34] Cooke AL, Morris J, Melchior JT, Street SE, Jerome WG, Huang R, Herr AB, Smith LE, Segrest JP, Remaley AT (2018). A thumbwheel mechanism for APOA1 activation of LCAT activity in HDL. J Lipid Res.

[35] Anson M, Crain-Denoyelle AM, Baud V, Chereau F, Couty J (2012). Oncogenic β-catenin triggers an inflammatory response that determines the aggressiveness of hepatocellular carcinoma in mice. J Clin Invest.

[36] Phesse TJ, Parry L, Reed KR, Ewan KB, Dale TC, Sansom OJ, Clarke AR (2008). Deficiency of Mbd2 attenuates Wnt signaling. Mol Cell Biol.

[37] Karlsson T, Vahlquist A, Kedishvili N, Törmä H (2003). 13- cis -Retinoic acid competitively inhibits 3 α -hydroxysteroid oxidation by retinol dehydrogenase RoDH-4: a mechanism for its anti-androgenic effects in sebaceous glands?. Biochem Biophys Res Commun.

[38] Zhu YH, Li JB, Wu RY, Yu Y, Li X, Li ZL, Zhang HL, Feng GK, Deng R, Zhu XF (2020). Clinical significance and function of RDH16 as a tumor-suppressing gene in hepatocellular carcinoma. Hepatol Res.

[39] Briones-Orta MA, Avendano-Vazquez SE, Aparicio-Bautista DI, Coombes JD, Weber GF, Syn WK (2017). Osteopontin splice variants and polymorphisms in cancer progression and prognosis. Biochim Biophys Acta Rev Cancer.

[40] Lamort AS, Giopanou I, Psallidas I, Stathopoulos GT (2019). Osteopontin as a link between inflammation and cancer: the thorax in the spotlight. Cells.

[41] Cabiati M, Gaggini M, Cesare MM, Caselli C, De Simone P, Filipponi F, Basta G, Gastaldelli A, Del RS (2017). Osteopontin in hepatocellular carcinoma: a possible biomarker for diagnosis and follow-up. Cytokine.

[42] Gasnereau I, Boissan M, Margall-Ducos G, Couchy G, Wendum D, Bourgain-Guglielmetti F, Desdouets C, Lacombe ML, Zucman-Rossi J, Sobczak-Thepot J (2012). KIF20A mRNA and its product MKlp2 are increased during hepatocyte proliferation and hepatocarcinogenesis. Am J Pathol.

[43] Maxwell CA, McCarthy J, Turley E (2008). Cell-surface and mitotic-spindle RHAMM: moonlighting or dual oncogenic functions?. J Cell Sci.

[44] He X, Liao W, Li Y, Wang Y, He S (2015). Upregulation of hyaluronan-mediated motility receptor in hepatocellular carcinoma predicts poor survival. ONCOL LETT.

[45] Jelluma N, Brenkman AB, van den Broek NJ, Cruijsen CW, van Osch MH, Lens SM, Medema RH, Kops GJ (2008). Mps1 phosphorylates Borealin to control Aurora B activity and chromosome alignment. Cell.

[46] Yang CH, Kasbek C, Majumder S, Yusof AM, Fisk HA (2010). Mps1 phosphorylation sites regulate the function of centrin 2 in centriole assembly. Mol Biol Cell.

[47] Liu X, Liao W, Yuan Q, Ou Y, Huang J (2015). TTK activates Akt and promotes proliferation and migration of hepatocellular carcinoma cells. Oncotarget.

[48] Pan SJ, Ren J, Jiang H, Liu W, Hu LY, Pan YX, Sun B, Sun QF, Bian LG (2018). MAGEA6 promotes human glioma cell survival via targeting AMPKα1. Cancer Lett.

[49] Yang H, Xiong FX, Lin M, Yang Y, Nie X, Zhou RL (2010). LAPTM4B-35 overexpression is a risk factor for tumor recurrence and poor prognosis in hepatocellular carcinoma. J Cancer Res Clin Oncol.

[50] Yang H, Xiong F, Qi R, Liu Z, Lin M, Rui J, Su J, Zhou R (2010). LAPTM4B-35 is a novel prognostic factor of hepatocellular carcinoma. J Surg Oncol.

