# Supplementary figures and images for: A robust twelve-gene signature for prognosis prediction of hepatocellular carcinoma
Source: Cancer Cell Int. 2020 Jun 3;20:207. doi: 10.1186/s12935-020-01294-9 (PMC7268417; doi:10.1186/s12935-020-01294-9)

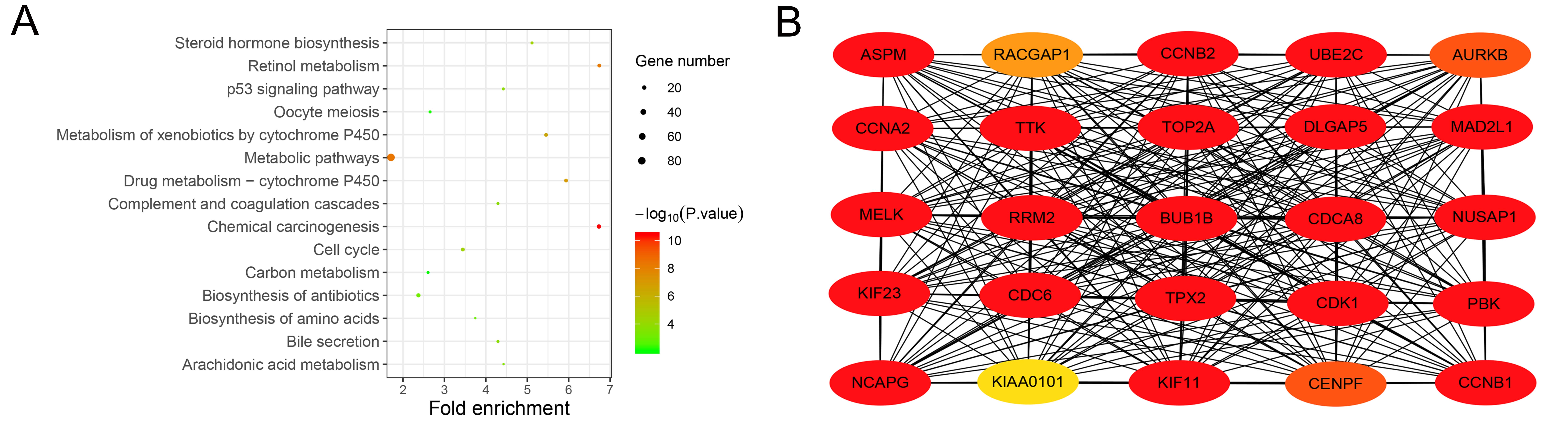

Supplement: Supplementary file 3 — Additional file 3: Figure S2. Functional analysis. (A) KEGG analysis of the DEGs; (B) the top 25 hub genes analyzed by the PPI network. [file 12935_2020_1294_MOESM3_ESM.jpg]

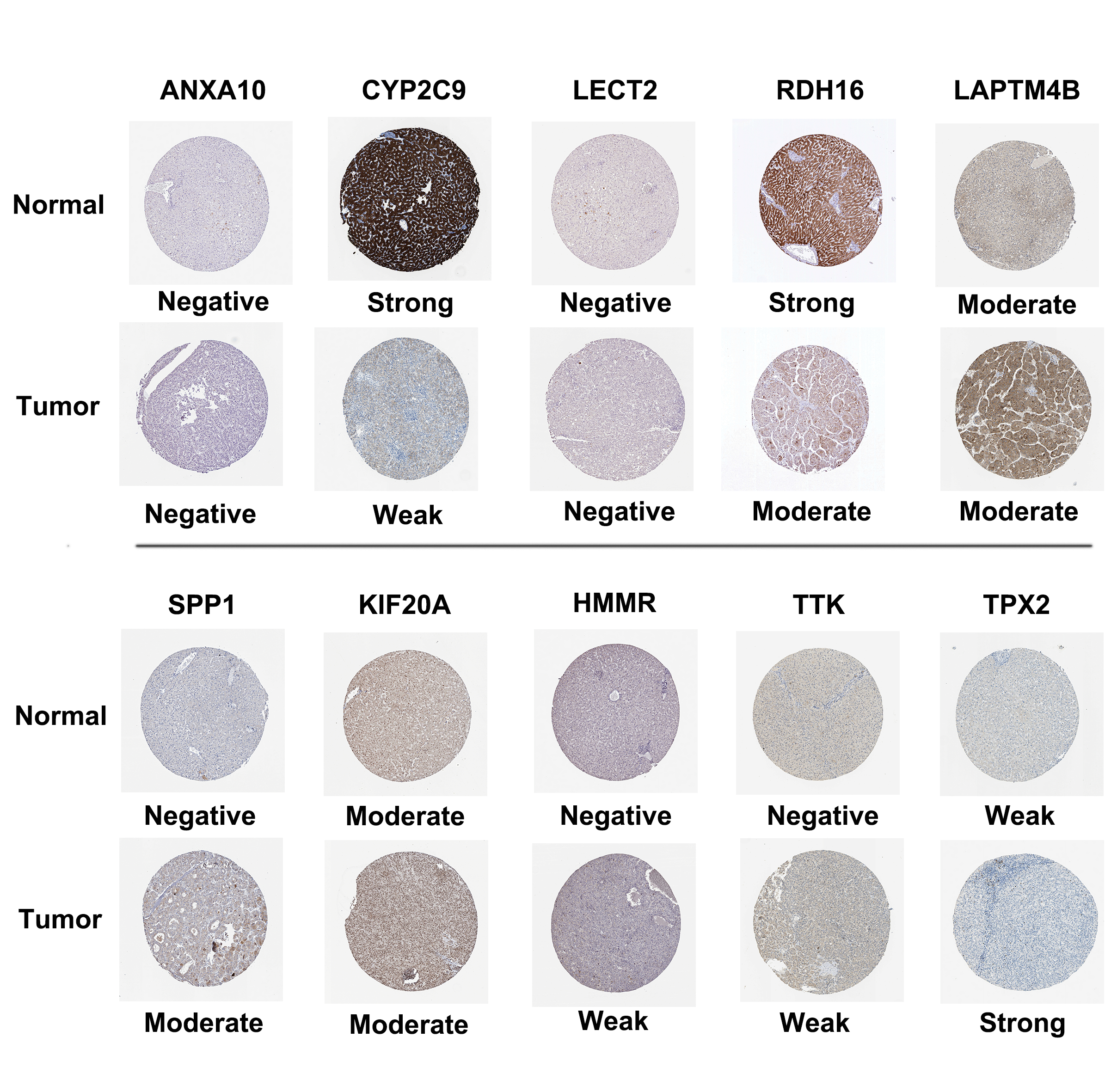

Supplement: Supplementary file 10 — Additional file 10: Figure S6. Typical IHC of twelve genes (except LCAT and MAGEA6, not included in the database) in tumor and normal liver tissues. [file 12935_2020_1294_MOESM10_ESM.tif]

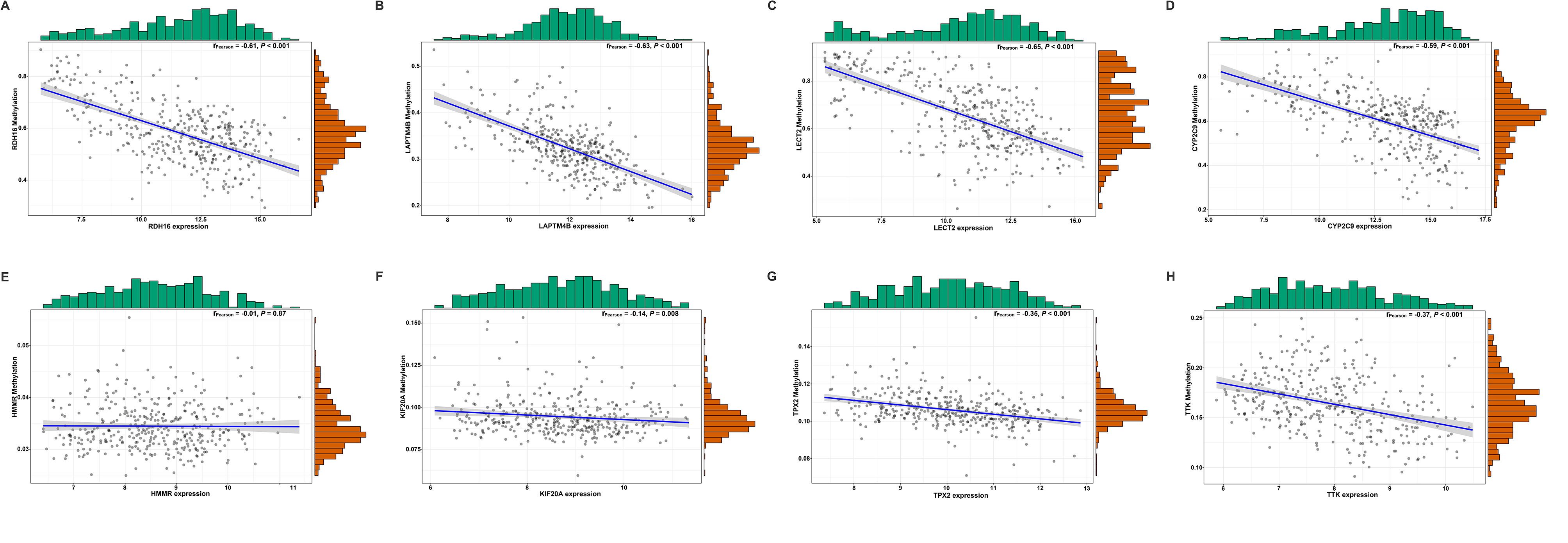

Supplement: Supplementary file 11 — Additional file 11: Figure S7. Regression analysis between eight gene expression and DNA methylation. [file 12935_2020_1294_MOESM11_ESM.tif]
